# Benefits from using recycling red blood cells in cardiovascular surgery

**DOI:** 10.1186/1749-8090-8-S1-O160

**Published:** 2013-09-11

**Authors:** R Almeida, M Steinbach, M Centenaro

**Affiliations:** 1 Cardiovascular Surgery, FAG, Cascavel, Brazil

## Background

Due to problems related to blood transfusion, decision by patients and the shortness of blood stocks, cardiovascular surgery is finding new paths, for not using homologous blood supplies. The authors will show if blood salvage (BS) is indicated in all patients submitted to cardiovascular surgery.

## Methods

We studied 77 consecutive patients submitted to cardiac surgery with use of BS and extra-corporeal circulation (ECC) from November 2010 to June 2012. All cases, except redos, were included and performed by only one surgeon. The sample was divided in three groups, depending on the time of ECC. In group A, the time of ECC was smaller than 45, in group B from 45 to 90 and in group C greater than 90 minutes. We analyzed the volume of red cells recovered and infused, the pre, intra and post-operative hemoglobin (Hb) and the number of packed red cells (PRC) units, which were transfused. The parameters to blood transfusion were Hb lesser than 8,0 or 9,0 g/dl whether the patient was hemodynamically unstable.

## Results

The average group´ age was 60,44 ± 12,09 years old, of whom 71,43% were males. The group A was formed by 5,19% of the patients, B by 81,82% and C by 12,99%. The volume of erythrocytes recovered and infused was respectively 1.360,50 ± 511,37 ml and 339,75 ± 87,71 ml in group A, 1.436,63±516,06 ml and 518,83 ± 183,0 ml in B and 2.137,00 ± 925,04 ml and 526,20 ± 227,1 5ml in C. About PRC transfusions, in group A 1,00 ± 2,00 PRC were transfused, in B 1,27 ± 1,85 PRC and in C 2,56 ± 2,01 PRC.

## Conclusions

That BS can be used in all patients submitted to cardiovascular surgery with ECC. However, it is only cost-effective in surgeries whose time of ECC is greater than 45 minutes.

